# State-Of-The-Art and Trends in CO_2_ Laser Cutting of Polymeric Materials—A Review

**DOI:** 10.3390/ma13173839

**Published:** 2020-08-31

**Authors:** Ray Tahir Mushtaq, Yanen Wang, Mudassar Rehman, Aqib Mashood Khan, Mozammel Mia

**Affiliations:** 1 Department of Industry Engineering, School of Mechanical Engineering, Northwestern Polytechnical University, Xi’an 710072, China; tahirmushtaqray@mail.nwpu.edu.cn (R.T.M.); mudassar@mail.nwpu.edu.cn (M.R.); 2 Department of Industrial and Manufacturing Engineering, University of Engineering and Technology, Lahore 54890, Pakistan; 3 Key Laboratory of High Efficiency and Clean Mechanical Manufacture, Ministry of Education, School of Mechanical Engineering, Shandong University, Jinan 266000, China; dr.aqib@nuaa.edu.cn; 4 Department of Mechanical Engineering, Khwaja Fareed University of Engineering and Information Technology (KFUEIT), Rahim Yar Khan 64200, Pakistan; 5 Department of Mechanical Engineering, Imperial College London, Exhibition Rd., South Kensington, London SW7 2AZ, UK

**Keywords:** polymeric materials, CO_2_ laser cutting, dross formation, assist gas pressure, cutting speed, kerf, cut quality

## Abstract

Carbon dioxide (CO_2_) laser cutting finds one of its most relevant applications in the processing of a wide variety of polymeric materials like thermoplastics and thermosetting plastics. Different types of polymeric materials like polypropylene (PP), polymethyl methacrylate (PMMA), low- and high-density polyethylene (LDPE, HDPE), are processed by laser for different household as well as commercial products in the industry. The reason is their easy availability and economical aspect in the market. The problems associated with laser cutting include heat-affected zone (HAZ) generated on the cut surface, kerf width (KW), surface roughness (SR), dross formation, and striations formation. Furthermore, other related problems include taper cutting for deep parts and high-power consumption. The primary purpose of this work is a comprehensive literature review in CO_2_ laser cutting of polymeric materials. The influence of parametric variation on the cut quality is also explained. Cut quality in terms of KW, SR, HAZ, dross formation, and striations formation is analyzed by optimizing cutting variables like laser power (P_L_), cutting speed (CS), assist gas pressure (P_g_), pulse frequency, nozzle type and its diameter, and stand-off distance (SOD). The effects of the laser cutting on the properties of different thermoplastics/thermosetting materials are also reported. However, this topic requires further studies on exploring the range of polymeric materials, and their optimal parameters selection to improve the cut quality. Therefore, the research gaps and future research directions are also highlighted in the context of CO_2_ laser cutting for polymeric materials.

## 1. Introduction

Conventional machining processes are able to process a large variety of materials. However, the complex profiles are difficult to be generated by these processes [1,2,3]. These processes also possess some restrictions to treat with difficult to machine materials. The nonconventional machining processes like electric discharge machining (EDM), electron beam machining, electrochemical machining, and abrasive water jet machining are also employed to achieve fine and accurate machining of materials. These processes can also generate complex profiles in difficult to machine materials.

An advanced technology named as laser beam machining is becoming the backbone of the materials processing industry due to its numerous applications and processes. Laser (an acronym for light amplification by stimulated emission of radiation) cutting can be performed by melting or vaporization of the material by melt shearing or by vaporization cutting. Laser cutting offers a highly precise cut quality by optimizing laser parameters to produce a little surface roughness (SR), and minimum heat-affected zone (HAZ). Laser cutting is also independent of material hardness. It offers more material utilization, no tool wear, and high cut quality [4,5,6,7]. Caiazzo et al. [8] presented an experimental study of process variables in carbon dioxide (CO_2_) laser cutting of polymeric materials such as polycarbonate (PC), polypropylene (PP), and polyethylene (PE). The effect of CO_2_ laser cutting of different polymer materials at different settings of laser parameters was analyzed and explained. It was proved that cut quality could be enhanced by using a low power CO_2_ laser.

A significant work is already available on the laser cutting of metals, composites, and alloys. Although, a significant breakthrough is also made in the laser cutting of polymeric materials over the last few decades. However, the laser cutting of polymeric materials is still needed to be explored further to enhance cutting efficiency. Therefore, this work is done to study the laser cutting of polymeric materials for a variety of household, and industrial applications. Numerous research studies on laser cutting have been reported to reduce HAZ, SR, dross and striations formation, and enhance material removal rate (MRR). The purpose of this work is a comprehensive literature review on cutting plastic polymers using a CO_2_ laser to understand and investigate the cut quality in terms of HAZ, kerf formation, SR, dross formation, striations formation, and material removal rate (MRR). Emerging challenges and future trends have also been proposed in the end.

## 2. Classification of Polymers

In general, macro-molecular compounds properties are strongly linked to changes in temperature [9]. Amorphous polymers at sufficiently low temperature are harder and glass-like, while at normal critical temperature they take a softer and moldable form, the glass transition temperature ($T_{g}$) [10]. At higher temperature $T_{g}$, polymer chain shows an increased variability that allows the flow of the bulk material. Flexibility is restricted at temperatures below $T_{g}$, and the polymer turns into a glassy, flexible, or elastic solid. $T_{g}$ is conditioned by its structural chemistry, physical or chemical interconnection, and molecular weight [11]. The $T_{g}$ value is considered as an important indicator of either a thermosetting or thermoplastic polymer’s classification of the plastic compound.

Synthetic polymers (like PP, PE) and semisynthetic polymers (like vulcanized rubber) have inherent material properties influenced by the values of the $T_{g}$ parameter that vary by molecular weight, as defined by Fox and Flory [12] with the relation reported in Equation (1).

(1) $$T_{g}\;={\;T}_{g}^{a}\;-\;\frac{K}{M_{n}}$$

Here,${\;T}_{g}^{a}$ is the maximum temperature at which the qualitative infinite molecular mass can be attained, *K* is an analytical value linked with the free polymer sample volume, and *M_n_* is its mean molecular weight [12].

### 2.1. Thermoplastic Polymers

Thermoplastic polymers like PE, PC, and PP, are the polymeric materials that, become softer and then harder after heating and cooling. When heating thermoplastic polymers to their melting point, they melt into a liquid. When these polymers are cooled below the $T_{g}$, they freeze to a glassy state. Thermoplastic polymers become soft and go into a viscous liquid state after reaching the $T_{g}$ or melting temperature. These polymers are then converted into glassy or semi-crystalline solids following the cooling method [13]. This process allows small cooling and heating cycles without any structure or function repercussions. For instance, shape and color altering, microstructural changes, and mechanical failure produce a reversible and restricted thermoplastic melting solidification behavior [14,15]. The whole crystal structure is changed if the heating temperature of the thermoplastic polymer is higher compared to its melting point. The linear molecular link chain dispersed randomly and changed special physico-chemical composition [16].

### 2.2. Thermosetting Polymers

Thermosetting polymers, also designated as thermosets, are irreversible plastic materials. The permanent changes can be made by heating over 200 °C. When a thermoset polymer heats up more than its melting point, the liquid–solid-state transition becomes irreversible, and it is called the solidification process. In the curing procedure, small molecules form a chemical bond, build complicated networks, and then establish a permanence of hard and rigid substance [13]. Similar to thermoplastics, the thermoset plastics are not temperature-dependent on the mechanical characteristics like tensile strength, hardness, and compressive strength.

## 3. Laser Cutting Mechanisms

The mechanism for the determination of a combination of material, assist gas, and laser type can be divided into three different categories which include vaporization cutting, fusion cutting, and chemical degradation.

### 3.1. Fusion Cutting

**Figure 1:**
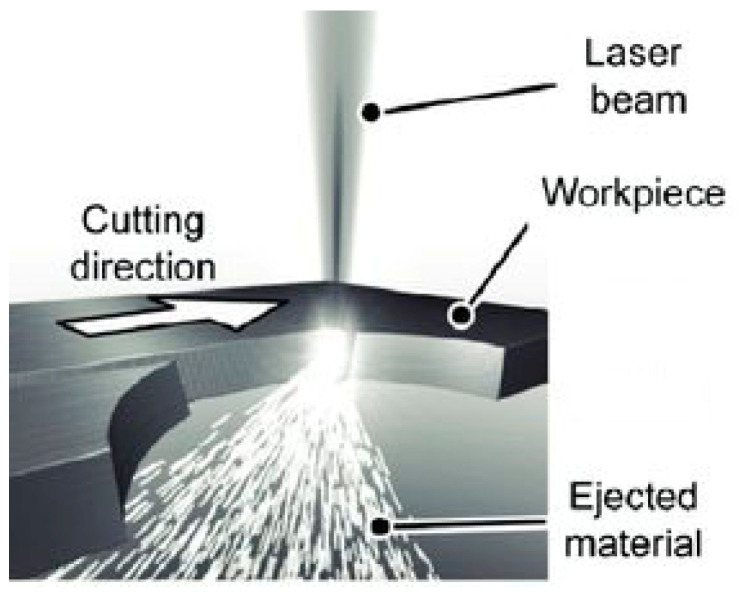
Fusion cutting mechanism [21].

Most thermoplastics are cut by melting the material, and this process is known as fusion cutting [17,18,19,20]. The underlying mechanism is similar to the inert gas metal cutting because it melts the material in-depth. The assist gas (usually compressed air) takes away the molten material, which is used to cut the workpiece. The kerf width (KW) varies between 0.2 mm and 0.8 mm depending on the material thickness. The cutting edge and the face are macroscopically smooth with certain streaks that are formed by the melt material from the laser beam from the point of entry to exit point. The materials that can be cut by fusion contain PE, PC, and PP [8]. The laser creates a small molten pool which is constantly blown away by the assist gas as can be seen in Figure 1 [21]. The cutting parameter level values for different polymeric materials are reported in Table 1.

### 3.2. Vaporization Cutting

**Figure 2:**
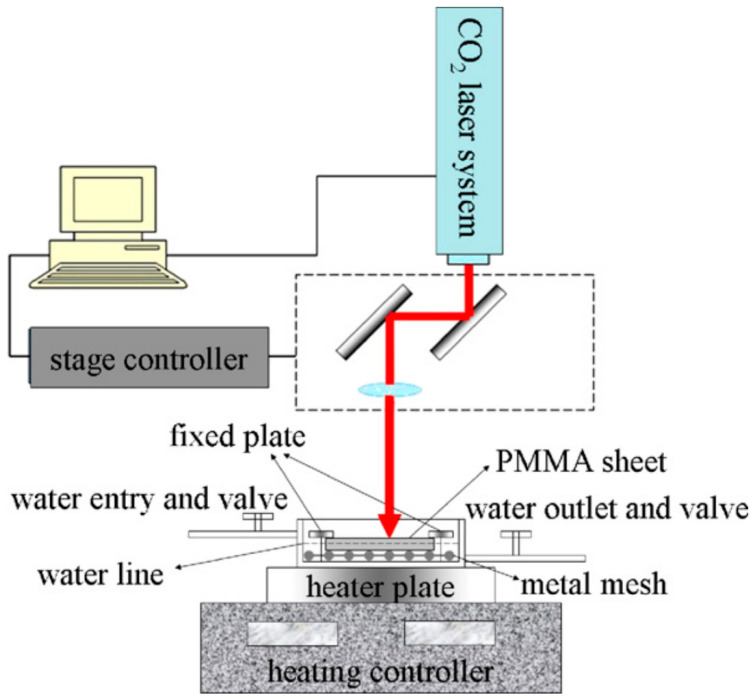
Polymethyl methacrylate (PMMA) laser cutting using vaporization [23].

In this type of cutting, the material is quickly heated to the temperature before significant thermal conduction melting occurs. Then, vaporization takes the material away and the fluid is normally ejected through the inert gas jet with the laser beam. The plastics mostly cut through the vaporizing mechanism include polymethyl methacrylate (PMMA), and polyacetal. An illustration of CO_2_ laser vaporization cutting is shown in Figure 2 [23]. The cutting edge resulted in high quality and produced a little kerf. This form is applied to thin portions since a unit quantity of material needs more energy to be removed [22,24]. The cutting parameters level values for PMMA material are reported in Table 2.

### 3.3. Chemical Degradation

The laser beam changes the material integrity by breaking chemical bonds in this mechanism, such as woodcutting. Woodcutting reduces big cellulose molecules to the main components of carbon as well as water vapor. The chemical degradation mechanism is used to cut a variety of thermosetting polymers such as rubber and epoxy resins. The mechanism generates a smooth and flat cut edge. The edge quality of cutting materials in chemical degradation is higher compared to the mechanical actions (conventional cutting) because it is soft and free of scattering. However, the cutting edge in this mechanism is covered with residual carbon layer dust that may require cleaning [22,24]. The cutting parameters levels for different thermosetting polymers are given in Table 3.

## 4. Quality Characteristics of Laser Cutting Edge

The accuracy in laser cutting is highly dependent on its quality characteristics such as KW, SR, dross, charring, and width of HAZ. The quality characteristics of the laser cutting are highly dependent on the input parameters, and their levels. The following section discusses these key features to address the cut quality (also known as quality characteristics).

### 4.1. Kerf Formation

**Figure 3:**
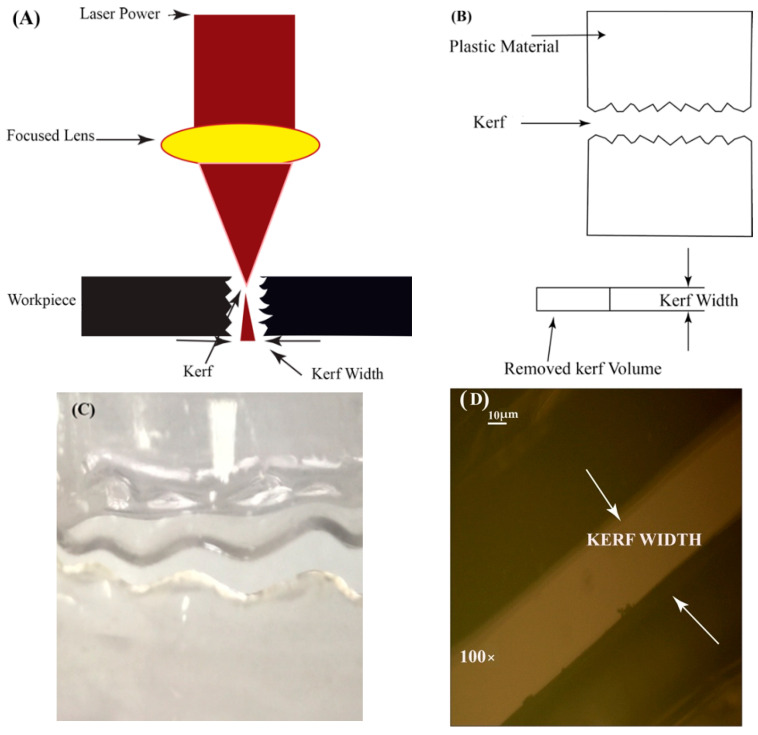
Schematic illustration of the cut material and its kerf formation (**A**,**B**): Schematic illustration of kerf and kerf width, (**C**): Physical representation of KW, and (**D**): Microscopic analysis of kerf width.

Kerf is defined as the slot produced as a result of material erosion and KW is the measurement of the excessive material removed (width of the cut slot) [25]. Thus, the slot produced by the laser is called kerf, and the width of that slot is called KW. The illustration of KW is shown in Figure 3. Eltawahni et al. [26] studied the kerf properties for PMMA and classified them as upper, middle, and lower kerf. Yilbas et al. [27] suggested a scaling law method to analyze the parametric trends for KW. It was concluded that both P_L_ and KW are directly proportional to each other, while CS is inversely proportional to KW by keeping P_L_ constant. KW is increased by increasing the workpart thickness. The focal point position of the lens caused an increment or decrement in the KW of material. Mathematical modeling is used to find an optimized value of KW and the model revealed the correlation with the measured KW of the PMMA material at low P_L_ [28]. Sulaiman et al. [29] explained the influence of different process variables like P_L_ and CS on KW in laser cutting. It was inferred that P_L_ and CS are highly significant for KW, and high-power lead to a wider kerf formation. Hossain et al. [30] explored the behavior of different process variables on kerf formation and resulted that stand-off distance (SOD), CS, P_g_, and P_L_ are highly influential parameters for KW. Gross et al. [31] reported the difficulties in understanding of a melting flow layer such as high temperature, intensity, P_g_, and dense structure of the kerf. An enhancement in P_L_ increased the KW, whereas an increase in CS reduced the KW [32,33].

The physical model of the material and kerf are shown in Figure 3A. Pictorial representation of kerf and removed material is shown in Figure 3B. Figure 3C indicates the physical view of the kerf. The microscopic examination of KW for the PE sample at 100× magnification is shown in Figure 3D.

Tamrin et al. [34] examined the influence of P_L_ and CS on performance measures such as MRR, and KW for PC. As a result, CS varied inversely with the work part thickness, and directly with the MRR. The authors also inferred that upper KW was found 1.5 times larger as compared to bottom KW. They further noted that the cut quality varied directly with the P_L_ up to a certain threshold extent and then gradually reduced. 

### 4.2. Striations Formation and Surface Roughness (SR)

**Figure 4:**
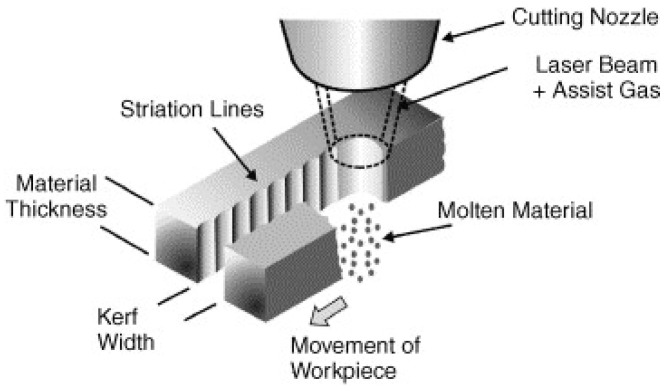
A schematic diagram for striation formation during the laser cutting process [35].

Striation formation is another quality characteristic of laser cutting surface. The surfaces that are cut with a laser beam are usually striated almost constantly resulting in a rough surface. Striations are not just produced by vibrations of the machinery. They appear in all the cases, independently of the laser workpiece displacement system vibrations. A schematic representation of striations formation is indicated in Figure 4. 

**Figure 5:**
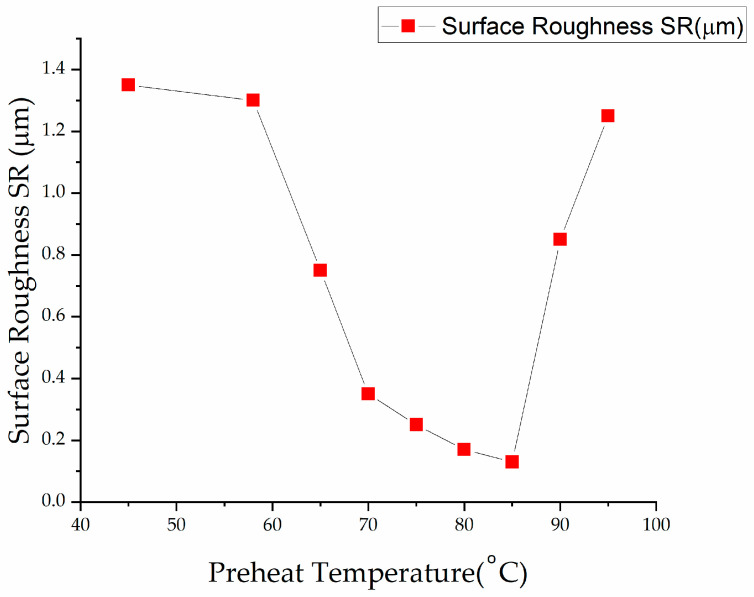
Graphical trend to show the preheated temperature effect on surface roughness (SR) [23].

The striation must be reduced to achieve optimized laser cutting, resulting in minimal SR [36]. The preheated temperature is needed to give a fine cut, and decreased SR in the case of PMMA plastic as can be seen in Figure 5 [23]. Yilbas et al. [37] conducted a comprehensive study to analyze striation formation and its influence on laser cutting variables. They inferred that the major reasons for striations formation include side-by-side burning, variation in absorbed power caused by surface plasma, and fluid layer surface oscillation.

### 4.3. Dross Formation

**Figure 6:**
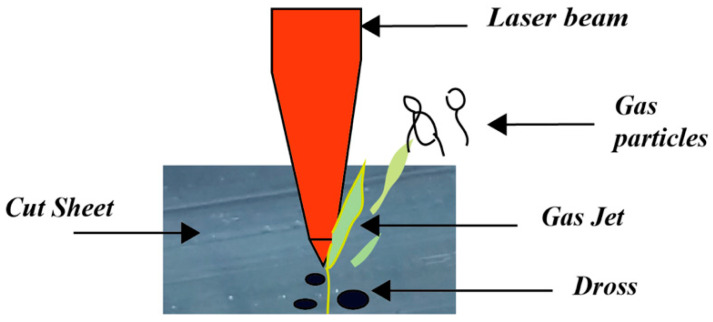
Representation of Dross formation during laser cutting.

The material stuck to the bottom of the work part after the laser cutting is called dross formation as demonstrated clearly in Figure 6. The dross may be solidified if the inert gas acts as an assist gas. Dross formation is dependent on the viscosity, and surface tension of the molten material. Materials with high viscosity and more surface tension resulted in more dross formation. 

Gas jet is used to eliminate dross from the underside of the part and mechanically removed after cutting [22,24,36]. Schuöcker et al. [38] conducted an experimental study on dross formation at the material edges as a result of laser cutting. As a result, dross and slag formation occur at the kerf wall not in the middle.

### 4.4. Heat-Affected Zone

**Figure 7:**
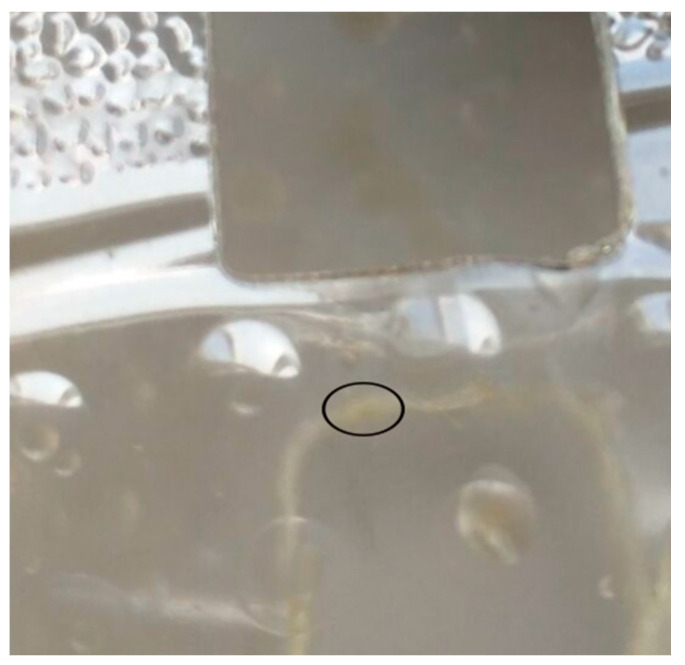
Heat-affected zone (HAZ) area of the laser cut material.

The HAZ is created during the laser cutting due to more localized heat and is the closed cutting zone (see Figure 7). Width size of HAZ is enhanced with the increase in energy input and cutting thickness for each unit length. The size of the width of HAZ is important to make cuts close to heat-sensitive sections. Besides this, it is not usually included in the laser cut quality evaluation [22]. Abrão et al. [39] reported the influence of different input variables to create a fine and effective cut in laser cutting. A proper experimental design was established to investigate the parametric effects. The burning of the workpiece occurs when the laser tool speed is less compared to its critical speed [40]. The thermal effects induced on the workpiece surface were reported by Ohkubo et al. [41] as examined by scanning electron microscopy.

### 4.5. Charring

Charring is defined as the chemical process caused by incomplete combustion during laser cutting. It is recognized by a black line in carbon-based materials. Therefore, the P_L_ should be controlled to avoid burning. Caiazzo et al. [8] examined the influence of laser cutting variables on charring formation. It was resulted that charring is reduced by increasing CS and decreasing P_L_. Akitt et al. [42] proposed an electronic supervisory system to improve power stability, and accuracy in laser processing. The P_L_ was measured after each quadrant of laser output for the calculation of its stability.

## 5. CO_2_ Laser Parameters for Polymeric Materials Processing

**Figure 8:**
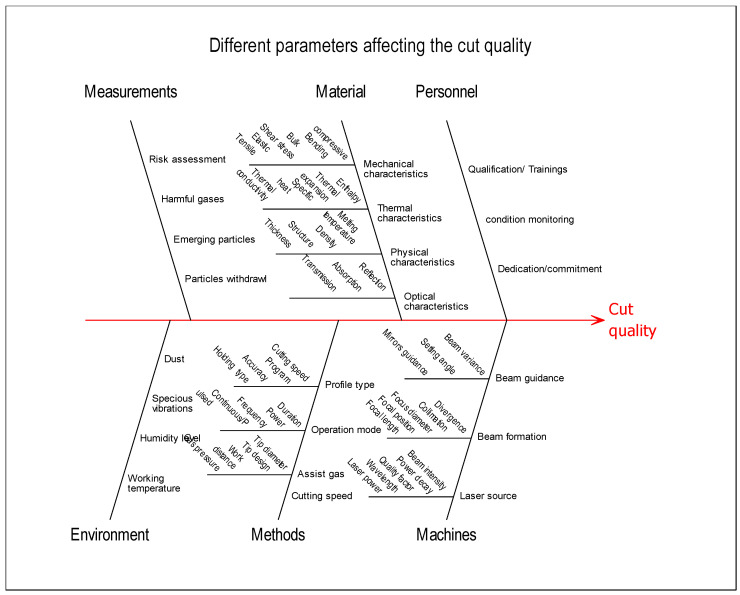
Cause and effect diagram to elaborate on the effect of different input parameters on output responses [44].

The characteristics of the work material, laser beam, the required KW, precision needed for the cutting process, and workpiece width are the main attributes on which laser cutting variables depend upon [43]. There are other various characteristics that directly or indirectly influence on the cut quality. A detailed illustration of different parameters affecting the cut quality is described in a cause and effect diagram as elaborated in Figure 8 [44].

**Figure 9:**
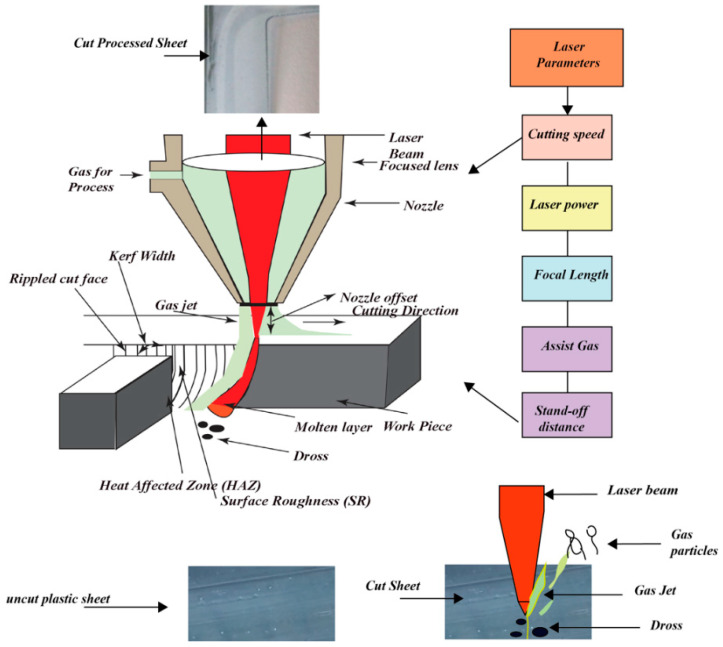
Detailed representation of the process of CO_2_ laser cutting, cutting variables, and output responses.

Kuo et al. [45] reported the comparison of low- and high-power laser in laser cutting. It was inferred that cut quality is significantly influenced by varying CS and P_L_ compared to other process input parameters. Figure 9 highlights some of the essential laser cutting variables.

### 5.1. Laser Cutting Speed

**Figure 10:**
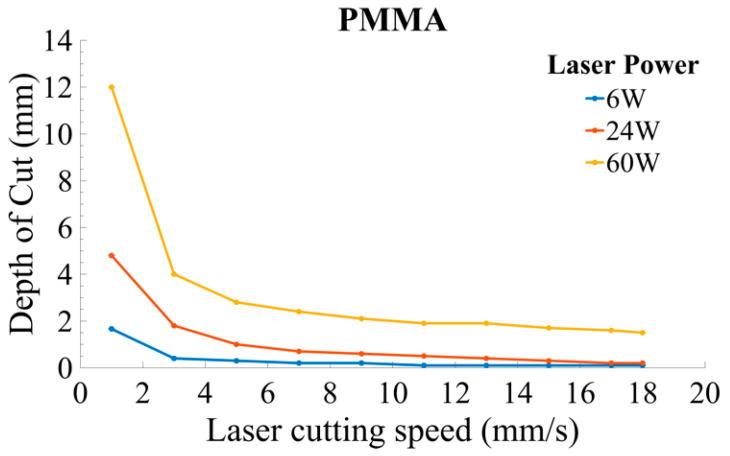
Parametric trends for CS and depth of cut (DOC) at different power ratings in PMMA [46].

The thermally affected region is reduced as a result of the increased CS. Therefore, CS should be kept high to minimize the thermal damages in the workpiece. The cut quality is highly influenced by CS. Depth of cut (DOC) varies inversely by increasing CS. Zhou et al. [46] derived the relationship of CS with DOC and P_L_ for PMMA material

(2) $$\mathrm{CS}\;=\;B\left[\frac{\alpha }{Q\;\times {\;R}_{b}\;\times \;\rho \;\times \;\sqrt{\pi }\;}{\left(\frac{P_{L}}{DOC}\right)}^{\omega }\right]$$

where CS symbolizes the cutting speed, P_L_ is the laser power, and α denotes the energy absorptivity. It is presumed that the laser radiation is entirely absorbed by material where α = 1. Q is the specific heat of the material, R_b_ = 0.25 mm describes the radius of the laser beam, ⍴ denotes the material density, and DOC depicts the depth of cut. B and ω are constants with the properties of the material. The values of B and ω have been checked from experiments. For different materials, B and ω have different values. Materials constant values used in Equation (2) are mentioned in Table 4. The expression reported in Equation (2) indicates that CS is varied directly as P_L_, and inversely with the cutting depth as depicted in Figure 10. The equation can be applied for practical reasons by the industries. They can use it to cut more depth material at low CS and low P_L_ or adjust according to the requirement.

**Figure 11:**
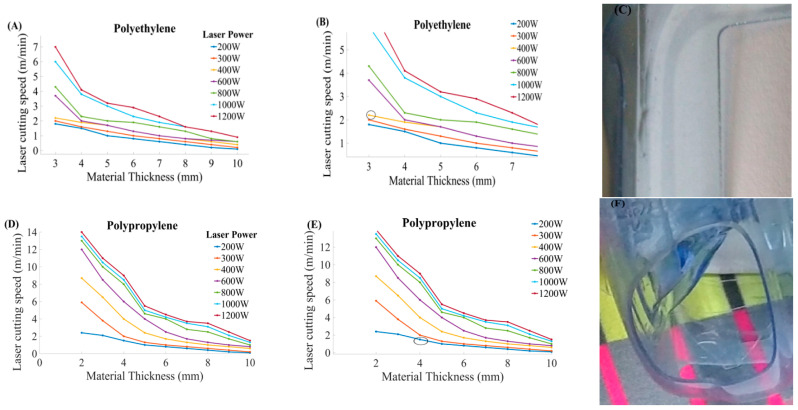
Parametric trends for CS and the thickness at different power ratings for PE and PP (**A**): Graphical trends for CS and thickness for PE at different power, (**B**): Illustration of steep curve to describe the relationship between CS and thickness, (**C**): Pictorial representation of PE cut part, (**D**): Graphical trends for CS and thickness for PE at different power, (**E**): Illustration of steep curve to describe the relationship between CS and thickness, and (**F**): Pictorial representation of PP cut part [8].

PE and PP material were cut at a different material thickness. It was observed that the thick workpiece needed high CS while the thin material needed less speed, as illustrated in Figure 11. Controlled CS is required to cut different thickness that leads to obtaining good cut quality.

Parametric trends for CS and thickness for PE at different power ratings as depicted in Figure 11A. It can be seen that CS is inversely proportional to the work part thickness. Figure 11B indicated the pointed part of Figure 11A. The CS for PE material was set at 3.8 mm/s, P_L_ of 300 W, along with the cutting thickness of 2 mm. The cut part is presented in Figure 11C. Figure 11D describes the parametric trends of CS and thickness for PP at various powers. Figure 11E indicated the pointed part of Figure 11D. The PP material with a 6 mm thickness was cut at 1.5 mm/s and 200 W P_L_. Figure 11F illustrates the pictorial view of the machined part.

1. Vishnulal et al. [47] performed an experimental investigation of CS, P_L_, and vibrational frequency (workpiece vibrations) for improving the laser cut quality in terms of MRR for PMMA, PC, and PP. The authors concluded that low-frequency vibrations ranging from 12 to 24 hertz (Hz) improved the cutting rate with more microcracks on the cut surface. Hashemzadeh et al. [48] found that CS is increased by increasing workpiece vibrations: a 12-Hz vibration frequency raises the CS from 0.2 m/min to 0.4 m/min. The proposed the two-pass fabrication method to increase MRR. It was concluded that various issues related to cutting like optimal parameters selection, dimensional variations, energy input, and HAZ width can be controlled by the careful optimization of CS, P_g_, and P_L_ using the two-pass fabrication method.
2. Nozzle diameter and CS are considered as the main factors for energy utilization effectiveness. An optimal parameter setting is required to utilize energy effectively [49]. The high temperature generated during cutting resulted in low cut quality because of the more HAZ produced. P_L_ and CS found significant variables to control the thermal effects of the work material [50]. Pietro et al. [51] analyzed the influence of CS on steady-state front temperature. The authors realized that both CS, and temperature are directly proportional to each other. It was further added that CS is increased because of heating edges during cutting.

**Figure 12:**
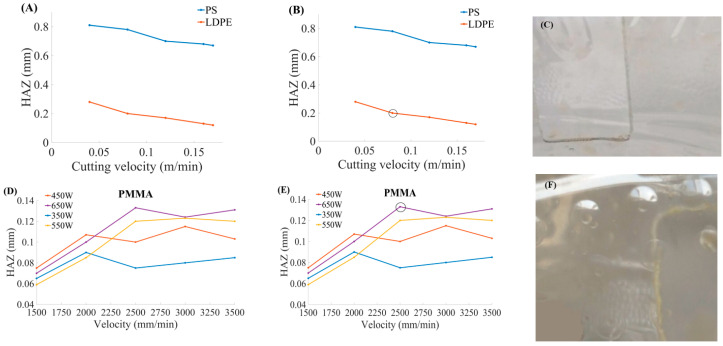
Parametric trends for laser CS, HAZ for PS, LDPE, and PMMA at different power ratings (A): Graphical illustration to show the influence of cutting velocity on HAZ, (**B**) Indication of pointed part of (**A**), (**C**): Pictorial representation of one of the cut parts among PS and LDPE, (**D**): Relationship between cutting velocity and HAZ at different power for PMMA, (**E**): Indication of maximum HAZ from (**D**), and (**F**): Pictorial representation of the cut part with maximum HAZ [52,53].

The HAZ is decreased by increasing CS but sometimes shows fluctuating behavior. There should be a controlled CS to minimize HAZ, and SR. The relationship of HAZ, and CS for PS, low-density polyethylene (LDPE) material [52], and PMMA material where it shows fluctuating behavior [53] as illustrated in Figure 12.

The HAZ was analyzed showing behavior for PS, and LDPE with respect to CS, as shown in the pointed part of the graph Figure 12B. Parametric trends indicated the relationship of CS, and HAZ for the PMMA material at different P_L_ values as illustrated in Figure 12D. The CS was found directly proportional to P_L_. The CS is increased with the increase of the P_L_. This material (PMMA) showed fluctuating behavior of the HAZ by varying CS, and P_L_. The machined specimen is shown in Figure 12F in a pointed zoom graphical representation from Figure 12E.

### 5.2. Laser Power

**Figure 13:**
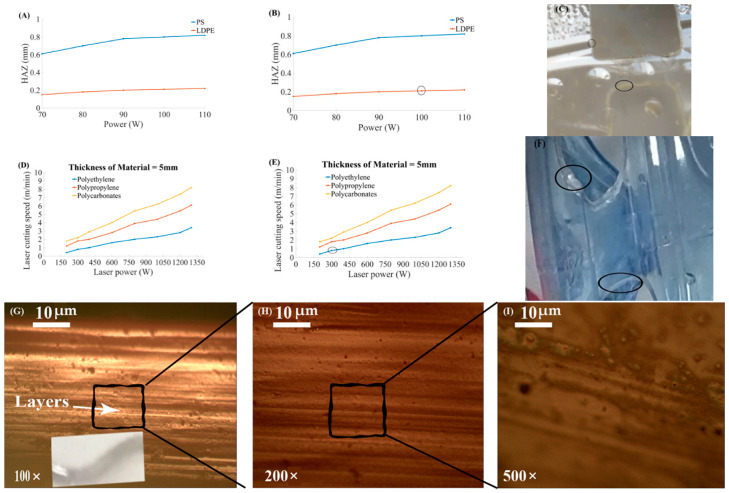
Parametric trends for P_L_ and CS for PS, LDPE, PE, PP, and PC at different power ratings (**A**): Graphical illustration to show the influence of laser power on HAZ for PS and LDPE, (**B**) Indication of pointed part of (**A**), (**C**): Pictorial representation of one of the cut parts among PS and LDPE, (**D**): Relationship between laser power and cutting speed at same thickness of 5 mm for PE, PP, and PC, (**E**): Indication of pointed part from (**D**), (**F**): Pictorial representation of one of the cut parts among PE, PP, and PC (**G**–**I**): Microscopic examination of cut part at 100×, 200×, and 500× [8,52].

The examination of thermal efficiencies has proved that low power leads to obtain a good cut quality. Usually, high P_L_ is employed for fast melting and ejection of work material during cutting. Stepak et al. [54] performed CO_2_ laser cutting of polylactic acid (PLLA) material for making tubular stents in surgical applications. Cut quality and geometry were studied by the changes in P_L_ and CS during machining. Sahin et al. [55] found that thermal efficiency is decreased by increasing P_L_. It was also found that the combination of high CS and less P_L_ lead to better thermal efficiency. Sahin et al. [56] explained the requirement of high thermal efficiency in the context of P_L_. Madić et al. [57] described the importance of optimal P_L_ in laser cutting. It was inferred that Taguchi and response surface methodology (RSM) are the vital tools to obtain the optimal results. Karimzad et al. [52] studied the influence of P_L_ and CS on cut quality in laser cutting of two different polymeric materials like LDPE, and PS. It was concluded that the combination of low P_L_ and high CS reduced the area of HAZ. It was also proved that the SR and tensile strength of the material are directly proportional to each other. The parametric trends for P_L_ and other different parameters are presented in Figure 13.

The relationship between P_L_ and HAZ for different polymers is described in Figure 13A. The HAZ is increased by increasing P_L_ [24]. According to this reported Figure, all materials indicated an increasing trend with HAZ, and CS by varying P_L_. LDPE exposed a significant variation in the HAZ. The response of the material is illustrated in Figure 13C. The zoomed graphical curve of the LDPE material is shown in Figure 13B. The material that showed a small HAZ at low P_L_ is evident in Figure 13C. Figure 13D indicated the parametric behavior of P_L_ and CS for different polymers. In Figure 13F, the 5 mm thick material was cut to examine the relationship between P_L_ and the CS. The zoomed part is shown in Figure 13E. The zoomed microscopic image of the cut edge is indicated to examine the cutting quality and the effect of the power on the plastic and resulted in a slight change of voltage creating layers on the cutting edge. In Figure 13G–I, scanning electron microscopy at 100× magnification indicated the layers on edge. It revealed the spots formation because of the dense focal spot heated thick focal spot. Such spots can also be formed when the ejected material touches the edge while cutting is ongoing. It needed optimal CS, and P_L_ to minimize the layers, and spots on edge to get finer cut.

### 5.3. Lens Focal Length

**Figure 14:**
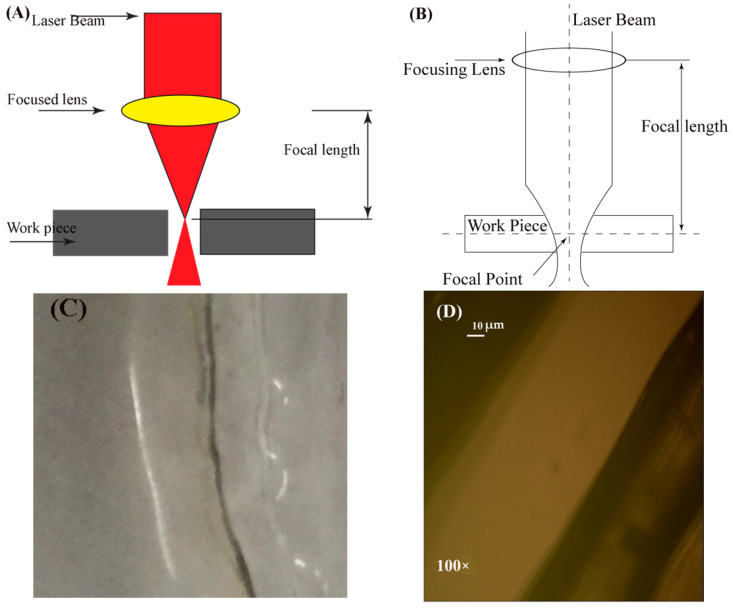
(**A**,**B**): Schematic representation of the focal point position/standoff distance in laser cutting, (**C**) Physical view of the cut part at more standoff distance, (**D**): Microscopic examination of cut part at high standoff distance.

The primary purpose of a lens is to converge and focus the light beam onto the work surface. The focal length of the lens can be adjusted by replacing different convex lenses (changing the lens curvature). The wider lens curvature gives short focal length, while the small curvature gives a large focal length. The point where the beam primarily starts cutting the material is called a focal point, as pointed in Figure 14B. This alteration in the focal point position significantly influences the cutting quality of the whole process. The cutting line is clear and transparent with a very little black line on the cutting surface at a short focal point distance Figure 14C. Nanoscopic examination of the kerf is presented in Figure 14D. A schematic illustration of the focal length of the lens in laser cutting is displayed in Figure 14A.

### 5.4. Assist Gas Pressure

The use of any assist gas in the laser cutting process has four main functions that impact cutting efficiency. These key functions include molten material ejection, back splatter protection of the lens, the cutting edge is refreshed, and an added heat source caused by a thermal exposure to active gas. P_g_ assisted in reducing dross formation and limiting the width of laser cutting, and HAZ [22]. The higher the Pg, the smaller the formation of dross and the lesser the HAZ. The degree of pureness of the assist gas is also affected by the cutting performance. A small quantity of impurity can reduce the total CS as well as increase the formation of dross. Compressed air is usually used in laser cutting because it is readily available for cutting plastics [24,36].

Man et al. [58] observed the supply pressure in particular for pressures over four bar. As a result, the lack of uniformity of effective jet pressure would lead to poor and inconsistent cuts, low cuts, and high gas waste. Man et al. [59] found that the flow of gas in the kerf is better if the P_g_ is reduced from 7 to 4 bar. It is because the more mass flow absorbs the kerf and without any significant difference, the gas flows along the kerf. The gas is easy to set up for thermoplastics: low pressure air. The pressure is usually increased for thermosets and composites, but high pressure causes the cut quality problems, i.e., charring. This problem is more apparent in thicker materials.

### 5.5. Nozzle Design and Diameter

It was concluded that P_g_ is highly influenced by the type of nozzle to improve cut quality. It was further explained that the cutting performance of the convergence nozzle is higher as compared to the tapered nozzle. Chen et al. [60] reported the demerits of high P_g_ on nozzle life. This problem was resolved by a device with a lens to enhance nozzle efficiency so that the nozzle can withstand high P_g_. A narrower nozzle diameter leads to a greater flow and the greater the amount of removal of molten material [59]. Riveiro et al. [61] pointed out the importance of finding new and cost-effective nozzle design to resolve degradation of the performance responses of the exit jet from conical nozzles currently in use.

### 5.6. Stand-Off Distance

The gap among the workpiece surface and nozzle for laser cutting is named as the stand-off distance. SOD is highly influenced by the flow pattern of the gas. SOD is usually estimated from 0.5 to 1.5 mm to reduce turbulence in laser cutting. A narrow stand-off distance provides a stable cutting environment irrespective of the risk of spreading the lens damage is increased [24].

### 5.7. Continuous Wave (CW)/Pulsed Beam Laser Mode

Continuous wave (CW) and pulsed laser beams are widely used in laser cutting, but typically CW is used. The laser cutting of plastics by the CW CO_2_ laser was studied theoretically and experimentally by Atanasov and Baeva [62]. Laser cutting of PMMA, Si-rubber, and Teflon-PMMA-Teflon structures was analyzed. A good agreement between theoretical estimation and experimental data was found. They pointed out that model relations, like the CS, can be predicted as a function of the substratum thickness or P_L_ and used for determining the optimal setting of the process parameters. Nylon was reported to be cut by either a CW or a CO_2_ pulsed laser. The process was optimized using a procedure called the 3D finite difference method. The quality of the edge can be improved significantly if pulsed laser mode is used [62]. Pulsed laser beam lower energy is usually chosen to precisely cut fine elements.

### 5.8. Pulse Frequency

When the pulse frequency is increased, the laser pulse overlap is consequently reduced then the energy of the individual laser input lead to decrease KW [63]. At this frequency, the 600 Hz pulse frequency, the kerf dross expulsion rate becomes nearly steady. It has been noted that the thickness and CS have an important effect on flatness. Laser cut quality can be enhanced by varying the combination of laser output intensity, and its pulse frequency [64].

## 6. Properties of Cut Material in CO_2_ Laser-Assisted Machining

Every material has its properties such as mechanical, thermal, physical, and optical characteristics, which influence the cut quality [65]. Some of these characteristics are briefly described here in the following subsections.

### 6.1. Mechanical Characteristics

The mechanical properties affected by the cut quality include elastic modulus, tensile/compressive strength, bulk modulus, shear modulus, and bending stress. The detailed examination of stress distribution provides an idea about the interactions of focal point position and stress concentration in the material. Goeke and Emmelmann [44] performed experiments for the investigation of the influence of materials properties on the cut quality. Radek et al. [66] performed the experimental investigation in laser cutting assisted modification by electrode spark deposition to analyze the surface microhardness, porosity, and adhesion. It was found that the aforementioned mechanical properties of the modified copper-tungsten electrodes are significantly influenced by the machined surface morphology. Their findings were validated through experiments.

#### Effect of Polymer Classification on the Mechanical Properties

**Figure 15:**
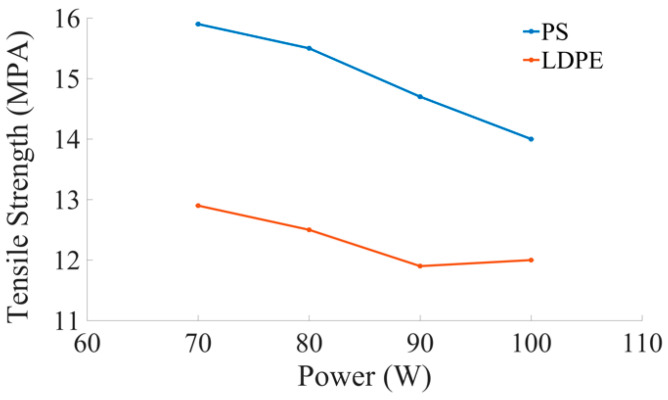
Parametric trend to indicate the effect of the power on tensile strength break for PS and LDPE [52].

The microscopic and mechanical properties of crystalline (like LDPE), and amorphous (like PS) polymers were studied in laser cutting [52]. The results indicated that:

- Microcracks, sink marks, and re-solid spots of molten material appear in amorphous polymers compared to the crystalline polymers. The microcracks were observed in three areas, such as cut surface, HAZ, and HAZ with base polymer boundary.
- HAZ and SR are decreased for semi-crystalline polymers.
- The laser input parameters affect both polymer types with almost similar effects on HAZ, SR, reducing P_L_, and increasing CS to improve the HAZ and SR.
- Semi-crystalline and amorphous polymers lost tensile strength after laser cutting. The formation of HAZ and SR are the reasons for this decrease in tensile strength.
- The presence of microcracks in HAZ plays a vital role in its tensile strength for an amorphous polymer.
- Among these two types of polymers, crystalline polymers result in better cut quality because of less HAZ and SR.
- The microcracks grow under the tensile load and result in sample fracture under the original tensile strength of the base polymer, according to microscopic studies that make PS cutting difficult.
- PS material’s tensile strength decreases significantly when power is increased as compared to LDPE material, as shown in the graphical illustration in Figure 15.

### 6.2. Thermal Characteristics

Thermal properties of the material such as thermal expansion, thermal conductivity, heat capacity, and enthalpy exert their influence on the cut quality. Among these properties, the thermal conductivity of a material is highly dependent on its morphology. The polymeric materials usually possess low thermal conductivity, which limits their use in high-temperature applications like in heat exchangers. The more the thermal conductivity of the polymer, the greater the temperature distribution, resulting in greater MRR [67,68]. Yilbaş et al. [69] established the relationship of temperature distribution with the work material thickness. The temperature distribution diminished when the size of the workpiece decreased. Banerjee et al. [70] made a comparison of the processing of various polymers such as fluoroelastomers (FKM), polyamide 6 (PA6) and thermoplastic elastomers (TPEs) in laser cutting. TPE contained less melted volume and area than PA6, whereas FKM possessed less melted area and volume among all previously stated polymers. The working temperature was found maximum on the workpiece surface and its edges. Choudhury et al. [71] conducted an experimental study in laser cutting, and concluded that HAZ varied directly with P_L_ and inversely with P_g_, and CS. The order of increasing HAZ produced for polymeric materials like PP, PMMA, and PC follows PP > PC > PMMA [53]. Thus, the cut quality of PMMA is higher compared to PC, and PP because of less HAZ produced on the cutting edge.

### 6.3. Different Strategies to Improve the Cut Quality

Various polymers like Nylon PA6-T27 result in poor cutting performance while processing using a CO_2_ laser. Accordingly, different methods were adopted to accelerate this cut quality like doping in 2.5–10% of Nylon using organic bentonite improved the cut quality [72]. Chiou et al. [73] implemented the six sigma principles to develop and understand the methods related to the properties of cut material. These principles were utilized in the laser cutting of acrylic polymers. The examination of different process parameters was made by using an internet-based machine vision (i.e., a camera with an image processing software), and Coordinate Measuring Machine (CMM). Modest et al. [74] developed a mathematical model to scribe the thickness of the material when studying the conduction properties of the material. The material’s inner temperature was predicted and measured after its initiation by laser. As a result, the formation of grooves and temperature is slightly influenced by the thermal effects of conduction and convection. Golyshev et al. [75] inferred that the amount of energy needed in laser cutting for obtaining the least SR in the melt flow is about 26 J/mm^3^.

## 7. Applications of CO_2_ Laser Cutting

Laser cutting plays a significant contribution to different industrial sectors for precision work. Stepak et al. [54] performed the CO_2_ laser cutting of PLLA material for making tubular stents in surgical applications. Ueda et al. [76] reported the benefits of laser cutting over conventional machining techniques in terms of work accuracy, fast cutting, and inexpensive system. The CO_2_ laser can assist in cost reduction and an increased production rate. In another study, Bonardi et al. [77] conducted the experimental study to decompose alkoxyamine through a CO_2_ laser. It resulted in the polymerization initiated for benchmark methacrylate monomers which considered an irradiated area, and having the significant potential for writing applications in 3D printing.

Additionally, CO_2_ laser processing eliminates distortion and increases the production rate, producing optimal microscopic results [78]. Sharp et al. [79] employed a CO_2_ laser for the manufacturing of medical types of equipment. Ink filler was used for showing a mark. Laser cutting is widely used to machine medical and surgical types of equipment. Therefore, it can also be regarded as a lifesaver. Caiazzo et al. [8] described some of the standard applications of laser cutting, which include cellular phones, orifice drilling, inkjet heads, flat panel annealing, optical circuits, drilling angioplasty devices, and catheters balloons. A parametric investigation on different laser types was performed, and as a result, the main application areas of laser cutting include material removal, cladding, and inscribing. Mayuet et al. [80] made a comparison of laser-assisted drilling with conventional drilling and stated that the laser processed part resulted in no mechanical vibrations, cutting forces and tool wear like in traditional drilling.

Wang et al. [81] proposed an idea for a laser marker to process it at low P_g_, and P_L_. Chryssolouris et al. [82] proposed a three-dimensional laser cutting approach for composite materials along with plastics polymers. The method involved the use of two interlinked beams for 3D cutting. These polymeric materials are used in daily life to manufacture plastic bottles. Stock et al. [83] reported the laser cutting process for CFRP material for its use in light material weight applications such as in aircraft and other instruments.

**Figure 16:**
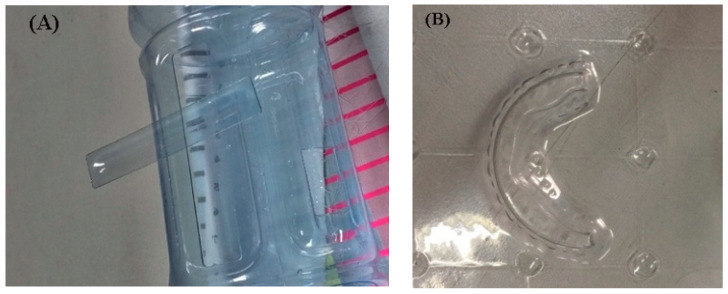
(**A**,**B**): Physical demonstration to show the laser cutting of polyethylene terephthalate (PET) at different parametric settings.

Schraft et al. [84] examined the laser cut quality by evaluating the dominance-free cancellation beam guidance component optical effects for obtaining high accuracy. The representation of a polyethylene terephthalate (PET) material cut by the CO_2_ laser is shown in Figure 16. Kim et al. [85] described the application of laser processing to join polymers. The authors found that the industries greatly benefited from the combination of PP, and PC when joined for household, and industrial products. However, the molecular power and adhesion forces of both PP and PC were found less. Rooks et al. [86] provided an example of three-dimensional laser cutting, which has typical applications in an assembly line of the automotive industry for cutting car sets and filling them with compounds. Genna et al. [87] reported the application of laser processing for making molds, and tools. It was found that the material’s cutting quality can be improved by clockwise cutting.

## 8. Conclusions and Remarks

The inspirations of using CO_2_ lasers in cutting different polymeric materials are undeniable for households as well as industrial applications. The present study is established to review the study of the influence of different process variables such as cutting speed (CS), laser power (P_L_), and assist gas pressure (P_g_), pulse frequency, nozzle type and its diameter, and stand-off distance for surface roughness (SR), heat-affected zone (HAZ), kerf width (KW), dross formation, charring, and striations formation, in the CO_2_ laser cutting of plastic polymers. The main findings of this work include:

- Low power CO_2_ laser cutting possesses a significant potential to improve the cut quality in terms of SR, HAZ, and KW, dross, and striations formation.
- The reported results indicate that: (a) SR is increased as P_L_ and CS are increased. (b) HAZ is varied directly with P_L_ and inversely with the CS. (c) KW is varied inversely with CS. (d) P_g_ is found highly influential in reducing the dross formation. (e) The control of P_L_ is considered as highly important factor to minimize the workpiece burning in terms of striations formation, charring formation, and HAZ. (f) Striation formation is significantly affected by the variations in P_g_, fluctuations in P_L_, and workpiece vibrations. (g) The polymeric materials with high density and surface tension result in more dross formation while laser cutting. (h) As P_g_ is increased, the dross formation and HAZ intensity is reduced. (i) Nozzle type and its diameter are highly influenced on P_g_ to improve the cut quality. (j) An optimized stand-off distance assists in stable cutting, and reduces turbulence. (k) As pulse frequency is increased, then KW is reduced.
- The properties of polymeric materials (thermoplastics/thermosetting) also influence on the cut quality in laser cutting. However, the mechanical and thermal properties are highly influential compared to other characteristics.
- Low power CO_2,_ laser cutting has shown promising results for almost all ranges of low melting materials. Therefore, it finds difficulty in processing high melt materials.

## 9. Shortfalls and Areas of Future Research

Previous sections were dedicated to elaborate the importance of CO_2_ laser cutting for polymeric materials, properties of these polymeric materials, laser cutting parameters, and different application areas. Significant work has already been done on CO_2_ laser cutting of polymers. Still, the following recommendations could be supposed to be the possible future research trends in laser cutting.

- Although the laser cutting variables like CS, P_L_, focal spot position, and P_g_ are comprehensively described, their combined influence on overall cut quality is still needed to be explored further
- Some of the quality characteristics like charring formation and striations formation in CO_2_ laser cutting are less significantly reported in the existing literature. Therefore, these should be further explored to improve the cut quality.
- The P_g_ imparts a significant role on the cut quality. Therefore, a suitable P_g_ range is needed to be calculated further by balancing the exact material requirements and the overall cut quality.
- As nozzle type and its diameter is highly influenced on P_g_ for cut quality. Therefore, the investigation on the design of the nozzle is yet needed to be explored further.
- The properties of polymeric materials like mechanical and thermal characteristics in the context of laser cutting are reported with an exploratory discussion. Other properties, such as optical characteristics including transmission, reflection, and absorption, are yet needed to be explored further.
- Different mathematical modeling techniques in laser cutting are yet needed to be explored to improve the cut quality.
- Some of the polymeric materials produce toxic environmental effects when burned; therefore, the environmental impacts of various thermoplastics/ thermosetting polymers in laser cutting are yet needed to be further elaborated.

CO_2_ laser cutting is found to be very useful in various industries, and household applications because of its consistently growing demand for a wide variety of low melting materials, especially plastic polymers. Therefore, it is still a potential research area for further investigation.

## Figures and Tables

**Table 1:** Cutting parameter levels for polyethylene (PE), polypropylene (PP), and polycarbonate (PC) in the fusion cutting mechanism [22].

Material	Thickness (mm)	Laser Power (P_L_, W)	Cutting Speed (CS, m/min)
PE	1	500	11
	3	500	2.2
	6	500	1
	9	500	0.5
PP	1	500	17
	3	500	4
	6	500	1.6
	9	500	0.9
PC	1	500	21
	3	500	5
	6	500	2.1
	9	500	1.1

Note: The assist gas pressure (P_g_) ranges from 1 to 4 bar, and the nozzle diameter ranges from 1 to 2 mm in fusion cutting.

**Table 2:** Cutting parameter levels for PMMA in the vaporization cutting mechanism [22].

Material	Thickness (mm)	P_L_ (W)	CS (m/min)
PMMA	1	500	35
	3	500	8
	6	500	3.5
	9	500	1.9

Note: P_g_ usually ranges from 1 to 4 bar for PMMA and nozzle diameters from 1 to 2 mm in the vaporization cutting mechanism. However, if the glossy edge is required, the P_g_ may be dropped below 0.25 bar.

**Table 3:** Cutting parameter levels for selected materials in the chemical degradation mechanism [22].

Material	Thickness (mm)	P_L_ (W)	CS (m/min)
Rubber	3	400	4
	6	400	1.6
	9	400	0.9
Rubber (carbon-filled, black)	3	400	3
	6	400	1.2
	9	400	0.35

Note: P_g_ usually ranges from 3 to 10 bar and nozzle diameters from 1 to 2 mm in chemical degradation.

**Table 4:** Properties values for different polymers [45].

Material	Density (kg/m^3^)	Q (kJ/g)	B	ω
PMMA	1180	2	0.415	0.7
Rubber	1300	2.1	0.791	0.83

## Nomenclature

CMM	Coordinate measuring machine
CO_2_	Carbon dioxide
CS	Cutting speed
CW	Continuous wave
DOC	Depth of cut
EDM	Electric discharge machining
HAZ	Heat-affected zone
HDPE	High-density polyethylene
Hz	Hertz
KW	Kerf width
LDPE	Low-density polyethylene
MRR	Material removal rate
PA6	Polyamide 6
PC	Polycarbonate
PE	Polyethylene
PET	Polyethylene terephthalate
P_g_	Assist gas pressure
P_L_	Laser power
PMMA	Polymethyl methacrylate
POM	Polyoxymethylene
PP	Polypropylene
PS	Polystyrene
Q	Specific energy of a material
R_b_	Laser beam radius
SOD	Stand-off distance
SR	Surface roughness
Tg	Glass transition temperature
TGA	Thermoplastic elastomers
t_i_	Interaction time
W	Watt
α	Energy absorptivity
